# Machine learning integrative approaches to advance computational immunology

**DOI:** 10.1186/s13073-024-01350-3

**Published:** 2024-06-11

**Authors:** Fabiola Curion, Fabian J. Theis

**Affiliations:** 1 Institute of Computational Biology, Helmholtz Center Munich, Munich, Germany; 2 Department of Mathematics, School of Computation, Information and Technology, Technical University of Munich, Munich, Germany; 3 School of Life Sciences Weihenstephan, Technical University of Munich, Munich, Germany

## Abstract

The study of immunology, traditionally reliant on proteomics to evaluate individual immune cells, has been revolutionized by single-cell RNA sequencing. Computational immunologists play a crucial role in analysing these datasets, moving beyond traditional protein marker identification to encompass a more detailed view of cellular phenotypes and their functional roles. Recent technological advancements allow the simultaneous measurements of multiple cellular components—transcriptome, proteome, chromatin, epigenetic modifications and metabolites—within single cells, including in spatial contexts within tissues. This has led to the generation of complex multiscale datasets that can include multimodal measurements from the same cells or a mix of paired and unpaired modalities. Modern machine learning (ML) techniques allow for the integration of multiple “omics” data without the need for extensive independent modelling of each modality. This review focuses on recent advancements in ML integrative approaches applied to immunological studies. We highlight the importance of these methods in creating a unified representation of multiscale data collections, particularly for single-cell and spatial profiling technologies. Finally, we discuss the challenges of these holistic approaches and how they will be instrumental in the development of a common coordinate framework for multiscale studies, thereby accelerating research and enabling discoveries in the computational immunology field.

**Supplementary Information:**

The online version contains supplementary material available at 10.1186/s13073-024-01350-3.

## Background

The immune system is a complex network of cells that co-orchestrate a defence response to unrecognized perturbations, that potentially endanger the host’s life. Throughout its evolution, this precise and lethal super-tissue has developed into a dynamic system capable of monitoring the environment in search of potential threats, via a scattered and highly specialized network of sentinel cells that constantly senses and reacts to infections and harmful changes in the organisms’ homeostatic equilibrium. The rigorous level of control that supervises its specialized activation is even more evident when, under a perceived threat, the immune system overreacts to pathogens or directs its attack against the host itself, eventually damaging healthy tissues and often degenerating into chronic diseases [1]. As immune cells are by design not bound to specific locations, they have adapted to operate in various microenvironments and under differing conditions of health and disease, making for an intriguing and constantly changing area of research. Studies have uncovered how the immune system plays an important role in complex diseases like neurological disorders [2, 3], diabetes [4] and cancer [5–7]. Given the involvement of the immune response in almost every aspect of an organism’s life, it is perhaps not surprising that there is considerable interest in leveraging the immune system to devise patient-specific immunotherapies [8–10].

The study of the immune system has focused on the evaluation of individual cells to identify characteristics that define their function. For a large part of the last two centuries [11], immunologists have built compendia of the cells that co-orchestrate immune responses by describing morphology, shape, tissue- and disease-specific occurrence and, more recently, phenotypic and molecular markers that delineate their lineage-membership and their evolution in time. Single-cell measurements are not new to immunology. Until recently, post-translational technologies measuring proteins like Fluorescence-Activated Cell Sorting (FACS) [12] and mass cytometry to single cells (CyTOF) [13] have been the preferred technologies to carry out individual cell characterization, thanks to selected surface or intracellular markers, whose expression levels are measured in thousands of single cells. However, with the advent of single-cell sequencing techniques, the field of immunology has advanced dramatically [11, 14–18].

The single-cell genomics revolution has impacted every field of biology and medicine [19]; in particular, the widespread adoption of barcoded droplet-based approaches [20] allowed a massive increase in the number of studies leveraging single-cell genomics. Since the launch of the international Human Cell Atlas (HCA) initiative [21], the global scientific community has leveraged single-cell genomics approaches to generate multiple atlases across tissues [22, 23], developmental stages [24] and diseases, with efforts such as the lung cell atlas [25] pioneering the field of integrative analysis of large collections of single cells. Similarly, recent studies have privileged the use of single-cell genomics to obtain a deep characterization of the immune system under healthy and disease conditions [19, 26, 27], such as infectious diseases [19, 26–28] a trend also boosted by the global COVID pandemic [29–31].

Recent technological advances in single-cell genomics allow measurements of multiple molecular read-outs: transcriptome, surface and intracellular proteome, chromatin, epigenetic modifications, immune repertoire and metabolites [32–34]. Both unspliced and spliced RNA transcripts are detected with standard scRNA-seq protocols [35], and furthermore, new protocols allowing lineage tracing, perturbation screenings with CRISPR-based transcriptional interference and sequencing of protein complexes [36] are offering new insights into dynamical properties of cells. Lastly, a series of spatial proteogenomics technologies have been developed, which combine microscopic imaging with gene expression [37], open chromatin [38] and proteome [39] while preserving the spatial location information.

In the past, scientists were used to analysing one modality at a time. Modelling the RNA expression provided a powerful means of identifying loci and genes contributing to disease [40]. Immune cell profiling was carried out with the help of a handful of protein markers in cell suspensions [41–43]. Likewise, the diagnosis of immune-mediated neurological disorders using structural imaging like magnetic resonance imaging (MRI) is non-trivial [44]. A typical immunology dataset could consist of several single-cell assays across multiple cellular readouts, bulk measurements, imaging, genetics and clinical data [45, 46]. As most phenotypes result from interactions where different biological layers are at play [47], multi-omics integrative studies provide comprehensive information on all these layers and unveil the hidden architecture behind a complex disease phenotype.

With the increasing size and complexity of these datasets, the future calls for approaches to generate comprehensive multimodal references onto which new datasets can be queried, to ensure fast knowledge transfer [48]. Integrating such multiscale datasets represents a new frontier for biomedical research. Broadly, the goal of machine learning (ML) integrative approaches is to generate a single representation of the various data sources, which can reduce the dimensions and preserve essential information from the input modalities such that the fused representation is more informative than the individual modalities [49, 50]. These embeddings form the foundation of the decision-making process: cell state identification, trajectory inference, molecular pathways and biomarker discovery and patient classification across complex collections of phenotypes.

**Fig. 1:**
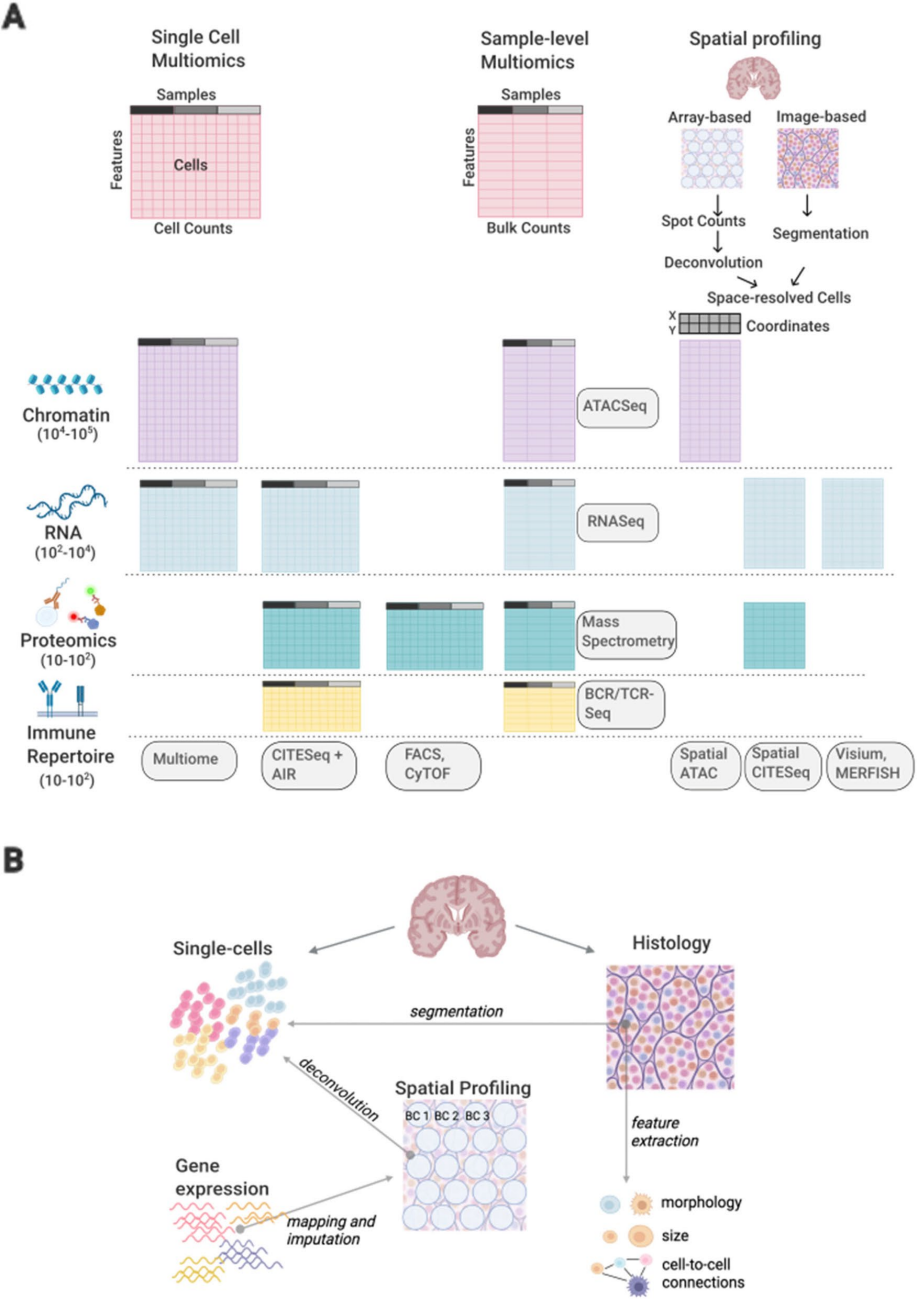
**A** Multimodal immunological datasets can comprise multiple assays across different modalities and resolutions. The number of features measured in each assay ranges from tens to hundreds of thousands. Some of these assays (CITEseq [59]; Multiome [60]) collect joint information from the same cells or samples (modalities aligned vertically). Different assays may share subsets of features (CyTOF [13], FACS [12]) (modalities aligned horizontally). Sample level measurements do not have a cellular resolution (ATAC-seq [61], RNA-seq [62], mass spectrometry [63]; BCR and TCR sequencing [64]) but can be performed in parallel to single-cell assays; spatially resolved cells can be extracted from multiple platforms, sequencing or imaging-based (Spatial ATAC [38], Spatial CITESeq [65], Visium [66], MERFISH [67]). **B** Spatial profiling carries information about RNA expression at individual spatial barcodes (BC). Single-cell references can be leveraged to deconvolute spatial data inferring cell type proportions and gene expression at spatial locations. Histological sections are often an accompanying assay. They can be segmented to recover cell and subcellular structures, as well as general tissue properties such as the morphology of cells, the density of cells at specific locations and cell-to-cell interactions

The data types of multi-scale datasets are various: generally, single-cell technologies consist of sparse matrices with rows and columns indicating cells and features (genes, proteins, chromatin regions), while spatial profiling techniques [51] provide subcellular or cell-aggregates molecular profiling, as well as accompanying images of tissues. Sample-level (bulk) omics measurements, genetic and clinical data do not have cellular resolution but suffer from missing values to different extents [52–56]. These techniques differ greatly in their resolution and consequently, data formats (Fig. 1). Others have covered the topic of integration [57, 58], classifying integration methods based on the relationship and type anchors across the modalities, and introducing terminology for the type of integration such as vertical, horizontal, diagonal and mosaic [57]. Datasets which include both paired and unpaired measurements from different omics require mosaic integration approaches, a type of integration complicated by the limited number or the total lack of shared features between the omics to align. In this review, we will briefly review some of the methods that have found applications in immunological studies, or hold potential for application in such contexts, highlighting key concepts for the integration (Additional file 1: Table S1). Given the increase availability of multiscale, unpaired datasets, we will highlight approaches to integrate these data. Finally, we discuss challenges and future development of integrative machine learning approaches, intending to inform those who intend to build up computational expertise to enable multimodal data interpretation.

## Integration of cell-based assays

Machine learning (ML) and multimodal integration are rapidly transforming immunological research by leveraging complex datasets from diverse sources [47, 57, 58]. Cross-technology integration of multimodal single-cell assays, such as CITEseq [59], with cytometry assays (FACS, CyTOF) can enable researchers to compound the domain knowledge accumulated with traditional proteomic techniques, to generate information-rich and interpretable references for immune studies.

Some methods originally developed for unimodal integration [68] are useful for integrating this type of multimodal data [47, 69, 70]. This first class of methods relies mostly on traditional linear models and has found wide applicability thanks to the intuitive interpretation of the linear manipulation of the data. On the other hand, a growing body of methods relies on Deep Learning (DL) techniques [71, 72], responding to the increasing complexity of multimodal data. Finally, given our focus on immunological applications, we include in this section methods developed for the integration of adaptive immune receptors (AIR) sequencing data with gene expression. Designed to work on paired data, these methods provide a much finer understanding of the adaptive immune system compared to any unimodal approach.

### Linear models

Flavours of linear decompositions have been successful at mosaic integration by leveraging shared features across data modalities [57]. For example, *LIGER* performs integrative non-negative matrix factorization (iNMF) [73] on the shared features, to distinguish between omic-specific factors and shared factors, followed by the construction of a neighbourhood graph using only the shared factors. By including an unshared metagene matrix [74] to inform the factorization, the authors were able to improve the integration of unmatched data across several platforms. *UINMF* [74] extends the LIGER model by accounting for unshared features between the modalities.

*CCA* is a popular dimensionality reduction [75], identifying canonical covariate vectors that capture sources of variance that are shared between omics that do not necessarily share features [76]. The CCA values can be used to identify cells, or “anchors”, with mutually similar profiles between the modalities, and correcting any systematic differences in expression levels between cells ensures their alignment. In [77], the authors leverage CCA to identify a rare subpopulation of CD11c-positive B cells, increasing upon COVID-19 infection, by integrating CyTOF and scRNAseq.

The same dataset is also used in *Bridge integration* [78]. With this approach, the authors characterized a very rare population of innate lymphoid cells, which were not identified in the CyTOF dataset, but correctly exhibited a CD25 + CD127 + CD161 + CD56 − immunophenotype. In this method, a multi-omic dictionary dataset is used as a bridge to translate between two experiments (the reference and the query) that have unpaired cells and features, but each share features with one of the individual assays of the bridge dataset. Horizontal integration of matching assays followed by a matrix factorization step allows generating a new set of shared features. Finally, these matrices undergo dimensionality reduction via Laplacian eigendecomposition and can be horizontally integrated.

*CyCombine* [79] integrates spectral flow cytometry, mass cytometry and CITESeq. After modality-specific preprocessing, which includes normalization or z-scaling of the expression of every marker in every batch, CyCombine clusters the cells into self-organizing maps (SOM) and applies a per-cluster batch correction method [80] to align the data and minimize technical noise. The authors were able to identify a relevant set of T and NKT cells increased in chronic lymphocytic leukaemia patients, and used the newly generated multimodal embedding to identify a PD-1-positive subset of CD8 + and CD4 + effector memory T cells, classically associated with CLL progression. Nearest rank neighbours (*NRN*) is a method to project flow cytometry data on an ABseq (Surface proteins + Transcriptome) reference [81]. After scaling protein expression within each assay, Euclidean distances between the FACS cells (query) and the reference data are calculated, and then, the query cells are projected onto the reference by k-nearest neighbours search following the scMap [82] batch correction method. Mapping the functional data obtained by flow cytometry onto the genomics space, the authors inferred differentiation dynamics of the haematopoietic lineage differentiation, recovering an early primary erythromyeloid versus lymphomyeloid split.

*MARIO* [83] matches cells across modalities by performing pairwise matching based on shared features and then projecting the distinct features using CCA. The matching is refined using a convex combination of initial and refined matchings in the CCA space. This method can perform integration across several proteomics assays (Cytof, CODEX [84]) and RNA. The authors were able to generate a cross-species blood atlas under challenged with the influenza virus and Interferon (IFNγ) and to correctly identify key populations of macrophages that sustain the recruitment of immature neutrophils in a COVID-19 lung dataset. Other relevant methods include optimal-transport-based *SCOT* [85], *PAMONA* [86] and *Stabmap* [87], based instead on mosaic data topology (MDT) network, with nodes corresponding to each assay, and edges weighted by the number of shared features between them. *MATCHER* [88] assumes one underlying biological process generating a linear manifold of each modality, resulting in one distribution for each modality. It then aligns them by projecting these curves onto a reference line. Finally, *CoNGA* [89] was developed for the integration of the T cell repertoire of TCRs with gene expression. CoNGA aims to find a joint representation of TCR and RNA from the same cells by identifying the overlap between the similarity graphs constructed on each modality independently.

### Deep learning approaches

**Fig. 2:**
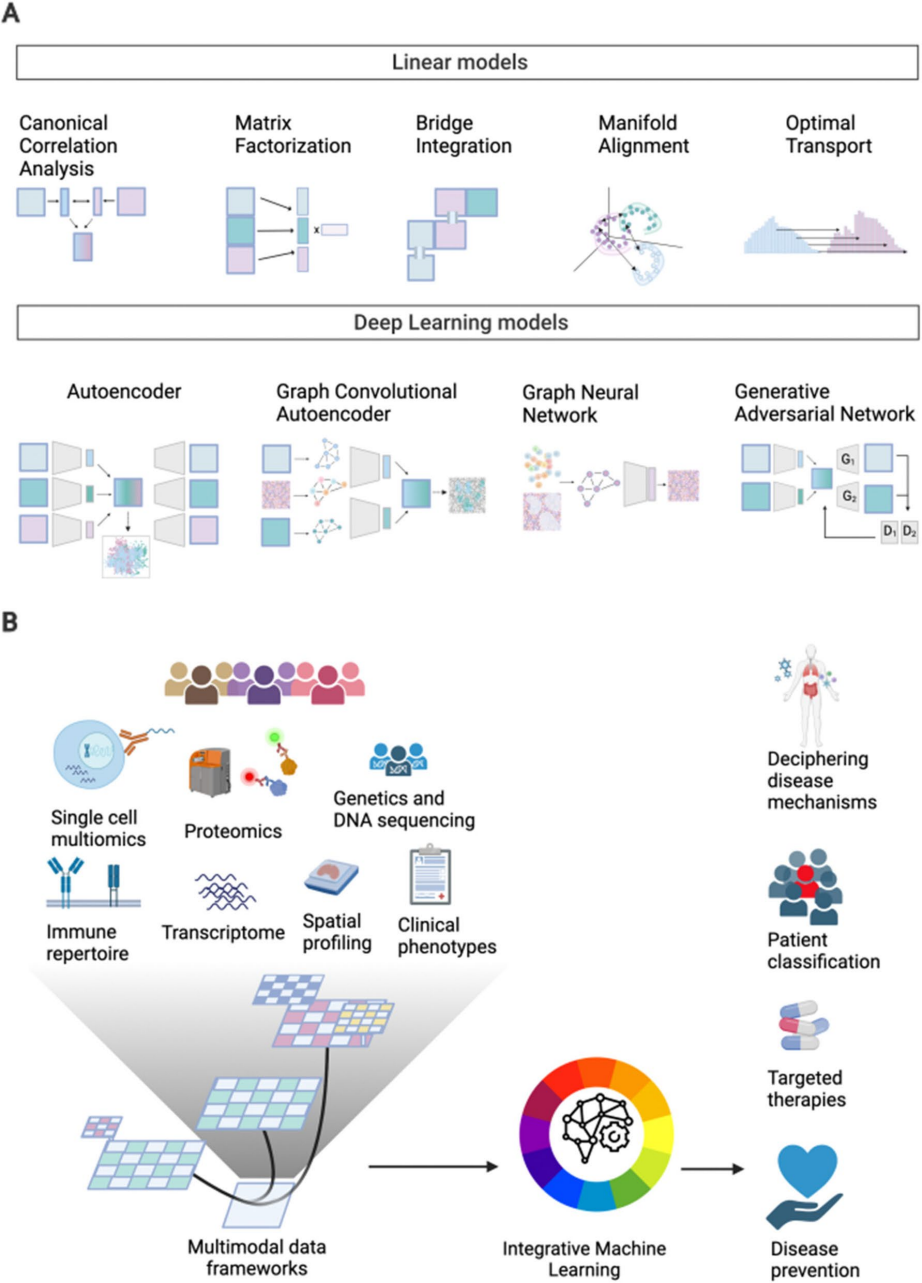
**A** Schematic diagrams of representative integration approaches for multimodal data. **B** Multimodal datasets have different data formats and can be stored in dedicated data structures that allow to efficiently access and process each layer independently or jointly. These data infrastructures sit at the core of machine learning integrative methods, which in turn provide diverse biomedical insights

One of the most widely used DL architectures for single-cell data is Autoencoders (AEs), neural networks that reduce dimensionality and/or noise from different types of data by combining an encoder and a decoder network [90, 91]. The encoder takes a raw data point from the input and maps it to a latent space of underlying factors, while the decoder controls the quality of the dimensionality reduction by reconstructing the original data from the latent representation. Variational Autoencoders (VAE) introduce Variational Inference to account for the irregularity of the latent space, returning for each encoded modality a distribution as opposed to a single point. Finally, the encoder and decoder can be distinct neural network architectures, such as Graph Neural Networks (GNN) [92], a type of neural network that can learn a latent representation from graph-structured data, or Generative Adversarial Networks (GAN) [93]. To align multimodal data, GANs train two neural networks, the generator (G) and the discriminator (D). The GAN model optimizes the integration by letting G and D compete against one another, respectively generating pseudo-data that resembles the real input, and discriminating between pseudo-data and real data (Fig. 2A).

*MAGAN* [94] aligns mass-spectrometry and scRNA-seq data, recovering shared immune cell populations in the presence of batch effects and enabling the imputation of proteins across the two technologies. MAGAN uses GANs to align data from different domains. Cells are mapped from one modality to the other minimizing a correspondence loss that measures the difference between points before and after the mapping. Autoencoders (AE) are a powerful architecture for multimodal data integration because multiple encoders can be used to learn efficient representations of the input data [95]. *TotalVI*, one of the first VAE frameworks proposed to integrate paired RNA and proteins from CITESeq assays, can be regarded as the reference method for more recent AE-based architectures tackling mosaic integration. TotalVI can denoise protein data and enable the classification of immune cells even in the presence of strong background antibody staining [96]. Some of the more complex VAE-based methods include *SCGLUE* [97], *Multigrate* [98] and *SCIM* [99]. In SCGLUE, the individual modalities encoders and a guidance graph that recapitulates features’ relationships across omics are encoded into related factors, which are then concatenated to form a unified embedding, using an adversarial alignment discriminator. scGLUE was demonstrated by integrating chromatin and RNA assays, recovering cis-regulatory events specific to monocytes and B cells [100]. Multigrate combines the single modalities distribution using the Product of Experts (PoE) to obtain the unified latent distribution, allowing the flexibility to deal with both paired or unpaired data. Multigrate allows atlas-level integration of multimodal PBMC datasets including COVID patients, providing a framework for atlas building, patient classification, feature extraction and biomarker discovery. In SCIM, the authors demonstrate the integration of scRNA and CyTOF cells from a melanoma dataset [101], aligning the individual modalities’ low-dimensional representations by simultaneously training the autoencoders and a discriminator network. The individual modalities’ latent spaces are aligned using an adversarial loss, resulting in a joint cellular latent representation. *Cross-modal autoencoders* [102] adopt unimodal AE to integrate and translate between data modalities, with a discriminative objective function to combine the data distributions from the different modalities in the latent space. If prior knowledge of matching features or cell types between domains is available, an additional loss term in the objective function will encourage the alignment between specific markers or the anchoring of certain cells. Integrating single-cell RNA-seq and chromatin images, the authors identified distinct subpopulations of naive CD4 + T-cells that are poised for activation. *MIDAS* [103], a recent deep generative framework for the integration of mosaic data, was demonstrated to disentangle two rare unconventional T-cell populations [104, 105] and successfully predict the differentiation trajectory of myeloid cells in the bone marrow [106] from the inferred embedding. scBridge [107] and scJoint [108] leverage scRNA labels to annotate and integrate multimodal datasets, both developed to integrate ATAC and RNA. scBridge iteratively computes and aligns prototypes of unlabeled scATAC and labelled scRNA cells, gradually aligning batches of cells in the latent space for the two modalities. scBridge uses a confidence score to highlight novel cell types and allowed to distinguish a subset of naive CD4 + T cells that were not detected with scJoint in a T-cell stimulation multimodal dataset [109]. scJoint treats the integration as a domain adaptation problem, constraining the labelled and unlabeled cells to align in the latent space by minimizing a cosine similarity loss, and further optimizing the integration with joint training after label transfer by kNN. The scJoint embedding allowed the authors to distinguish a small population of natural killer T cells in the same multimodal dataset [109].

Finally, three methods use DL frameworks for the integration of AIR with gene expression. *Tessa* [110], a parametric Bayesian hierarchical model, takes as inputs the single-cell RNA and the embeddings of the TCR obtained by feeding a numerical representation of the TCR amino acid sequences to an autoencoder. This latent representation summarizes clusters of related clonotypes which are then correlated to the gene expression profile clusters. Similarly, *Benisse* [111] learns a sparse weighted graph from the embedding of the high-dimensional data of BCRs, under the supervision of gene expression, so that BCRs closer to each other in the latent space have similar BCR sequences and represent B cells with similar transcriptomic features. Finally, *mvTCR* [112] is VAE generating a joint representation from TCR and RNA data. This tool is currently the only one with single-cell resolution on the TCR clonotypes variation, taking as input the RNA and the amino acid sequence of the alpha and beta chains of the TCR from individual cells.

## Methods for the integration of cells with spatial profiling data

Single-cell suspension sequencing methods have been largely successful within immunological research because most of the immune cells are not anchored in tissues and are therefore relatively easy to isolate. However, knowing the exact localization of immune cell compartments within solid tissues is important to elucidate cellular cross-talks in several diseases [113]. In cancer research, spatial molecular phenotyping of tumour micro-environment allows uncovering of mechanisms of immune escape [14, 114–116]; in autoimmune diseases affecting the central nervous system such as multiple sclerosis, failure of trophic and anti-inflammatory cellular communication was identified as clear features of the early stages of neurodegeneration [117].

Spatial profiling technologies capture biological patterns emerging in their context [51, 118, 119]. The landscape of spatial profiling methodologies is rapidly expanding [51] with applications in a variety of biological problems, including functional analysis of organs in multiple species, developmental processes and diseases [120, 121].

A distinction can be made across spatial techniques: (i) sequencing-based techniques for whole genomes, which do not have single-cell resolution [122, 123]; (ii) in situ sequencing techniques, which have increased resolution but smaller feature panels [124, 125]; (iii) in situ hybridization-based methods, which image smaller panels of RNAs or proteins at subcellular resolution, without sequencing readouts [38, 39, 66, 84, 126–132].

For sequencing-based methods, integration with single-cell data is needed to achieve at least two goals: prediction of the spatial distribution of features’ expression, especially when the spatial profiling technique has low coverage, and deconvolution of multi-cellular spots into cell types [133]. Lastly, methods for the integration of scRNA or Protein datasets with imaging-based techniques are also gaining popularity: most require segmentation of images into cell-readouts, and extraction of informative morphological features linked to individual pixels, while other segmentation-free methods discover spatial domains by reconstructing spatial organization of features in local neighbourhoods (Fig. 1B).

### Predicting spatial distribution of feature expression

Some of the methods employed for single-cell integration described in the previous section have been successfully adapted to integrate spatial transcriptomics with single-cell data [38, 73, 74, 76, 83]. However as spatial profiling techniques gain in popularity alongside single-cell protocols, ad hoc methods have started to emerge, and best-performing methods that integrate spatial data with single cells are often based on DL architectures [134].

Building on the MAGAN architecture, *scMMGAN* [135] can integrate ST and scRNAseq and can recover spatial expression of genes associated with breast cancer progression [136]. *Tangram* aims to maximize the spatial alignment of scRNAseq onto a spatial reference using non-convex optimization in order to retrieve a probabilistic distribution of the feature expression in the spatial data [125]. Interestingly, despite the overall good performance in the experiments shown, Tangram struggled to spatially map immune cells when dealing with different cell-type compositions between the cell suspension and the ST assay, or divergent expression profiles in a cross-species integration.

*gimVI* [137] is based on the scvi framework [90] and uses a generative model to infer the spatial distribution of undetected transcripts. *stPlus* [138] learns a joint embedding of the spatial transcriptomic data and reference scRNA-seq data via an autoencoder, to then predict the expression of spatial features based on the cell embedding via a weighted k-nearest-neighbor (kNN) method. *SpaGE* [139] leverages the domain adaptation algorithm PRECISE [140] to align the sc and spatial datasets, computing linear latent factors on each dataset and finding gene combinations expressed in both datasets to obtain a joint representation of the data. On this embedding, a kNN algorithm is used to predict the expression of spatially unmeasured genes. OT [141] based methods, such as *novoSpaRc* [142], *SpaOTsc* [143] and *Moscot* [144], assume that single-cell suspensions can be mapped to a tissue space based on similarities between expression profiles. A probabilistic mapping that assigns each cell a distribution over locations on the physical space is computed, allowing to reconstruct spatial gene expression. Additionally, SpaOTsc uses the new coordinates to infer a cell-to-cell communication network based on patterns of ligand and receptor expression, and intercellular regulatory relationships between genes are reconstructed for each pair of genes at a given spatial distance.

### Cell type deconvolution of spatial data

To improve the resolution of genomic-scale spatial technologies, methods were developed to estimate the abundance of given cell types at individual spots in histological sections. Many require an annotated scRNA-seq dataset with known cell-type markers to deconvolve the spatial data, adopting similar concepts applied for the deconvolution of bulk-RNAseq into single-cell profiles [145–147]. Indeed, methods like MuSiC [147] are often included in cell deconvolution benchmarks [148, 149], performing on par with methods designed ad-hoc for spatial data.

*Cell2location* [150] uses a reference single-cell dataset and the gene expression signature of the cell subpopulations in scRNA-seq data as input to infer gene expression at individual spatial locations into reference cell types. The authors were able to demonstrate cell2location ability to distinguish rare cell types, such as pre-germinal centre B cell population in a human lymphnode, and resolve the fine-grained immune cell types of the human gut. *RCTD* [151] employs supervised learning to estimate mixtures of cell types at each pixel. *SpatialDWLS* [149] first identifies the most likely cell types at each spot then a weighted-least-squares approach to infer cell type composition in the tissue.

*SPOTlight* [152], *DSTG* [153] and *CARD* [154] use the same pancreatic ductal adenocarcinoma (PDAC) dataset [155] with varying performances and results. SPOTlight was able to recover tumour-specific immune cell states in PDAC, applying a seeded non-negative matrix factorization (NMF) to obtain cell type-specific factors or topic profiles, then non-negative least squares (NNLS) regression is used to map each spot’s transcriptome to a topic profile and determine the weights for each cell type that best fit each spot’s topic profile by minimizing the residuals. Directly benchmarked against SPOTlight, DSTG additionally identifies spatial expression of marker genes associated with hypoxia and antigen presentation. DSTG leverages topological relations inside the data using graph-based convolutional networks to discover cell-type composition at spatial locations. CARD adapts the NMF framework to model spatial dependencies allowing a conditional autoregressive modelling on the columns of the inferred non-negative matrix. CARD could correctly infer global cell composition and gene expression of individual ST niches. *STRIDE* [156] trains a topic model on the scRNA-seq data to deconvolute cell types from spatial mixtures and was able to detect regulatory T lymphocytes at the interface between normal and tumour cells on a squamous cell carcinoma dataset [157]. *Stereoscope* [158] builds a probabilistic model to learn cell-type specific parameters on gene expression from scRNA-seq to obtain the cell mixtures in spatial data. *DestVI* [159] relies on variational inference to predict discrete cell-type-specific profiles and continuous latent variables of cell-states to describe the tissue architecture, and it is able to recover the interferon-induced changes in gene expression and cell-type composition of the spatial organization of lymph nodes upon bacterial infection. Recent methods can accomplish deconvolution without reference scRNA. *BayesTME* [160], a Bayesian generative model, can accurately infer cell type composition of ST data, identifying immune cells at the interface with tumour cells in melanoma samples. The *SpatialGlue* [161] framework does not use a modality as a fixed reference, but learns a spatial proximity graph and a feature graph from each modality which are then the basis for the shared embedding, introducing within and across-modality attention aggregation layers to account for modality-dependent contributions. With this new strategy, the authors can reconcile the mismatched modality-dependent cell type annotations, correctly identifying the spatial distribution of B cells and T cells, and subpopulations of macrophages in individual spatial niches from a spleen [39] and thymus [162] multimodal datasets.

### Integration of sc with imaging data

Integration of single cells with imaging data has the potential to complement the large body of histopathology and immunohistochemistry-based research with the mechanistic insights offered by single-cell sequencing data. After segmentation and quantification, images can be broken down into information that is complementary to single-cell sequencing data [121, 163] and integrated with standard approaches. For example, Tangram, novoSpaRc, SpaOTsc and Seurat’s CCA described before have also the capacity to assign cells from scRNA-seq data to spatial locations in histological sections.

Alternative segmentation-free approaches have emerged that leverage the structural properties of tissue images to discover the spatial domains emerging from networks of dynamically interacting cells. Spatial data provides additional information that goes beyond nonmolecular features of cell representations, including morphology, the density of cells at individual locations and differential cell-to-cell communication in heterogeneous tissues. Integration of these with uncoupled single-cell readouts is possible [102, 164].

Methods like *SpaGCN* [165] and *Spage2vec* [166] rely on graph representation learning, a powerful deep learning framework that leverages relational information retained in local cell neighbourhoods [121]. SpaGCN demonstrates how incorporating histology information can improve the detection of cancer regions on the PDAC [155] dataset, using the inferred spatial domain to recover spatially variable genes associated with the disease [167, 168]. Similarly, *STlearn* [169] infers dynamic trajectories of biological processes in spatial data by integrating histology and gene expression and was able to detect immunoregulatory cell-to-cell interactions in breast cancer samples [170]. *SpaCell* [171] integrates tissue morphology and gene expression data to perform cell-type and disease-stage classification.

Given that the vast majority of datasets available come from unpaired data and a mixture of matched and unmatched samples, we anticipate that methods leveraging self-supervised learning such as contrastive learning and multiple-instance learning will be useful to learn models of biological diseases from high-dimensional data.

## Methods for the integration of single cells within multiscale data collections

Datasets that result from collecting uncoupled modalities over time represent the largest body of data to analyse [172]. Strategies to integrate such datasets will need to make the heterogeneity a strength rather than a limitation. As single-cell datasets increase in size by profiling hundreds of patients, studies have now the power to link single-cell gene expression to genetic variation [173] or other data modalities profiled in bulk. In the context of integrating multiscale datasets, the difference in dimensionality and feature profiled represents the main challenge to obtaining a unified embedding [174]. The hierarchical nature of biological systems requires incorporating this structure in model design in the form of informative priors [175, 176]. Manifold alignment techniques could in principle provide an effective way of finding a low-dimensional embedding of multiscale data collections that preserves any known correspondences between them [177]. Similarly, strategies adopting cross-modal autoencoders can map heterogeneous data to a shared embedding and can learn holistic representations of cell and entire patient’s physiological states [102, 164]. Finally, linear models like tensor decomposition [178] can prove powerful for the identification of pathways that are connected to disease progression and highlight key biomarkers for pharmacological treatment [45].

When the same patients are profiled across multiple platforms, approaches that focus on integrating data leveraging the common sample axis by summarizing the single cell expression into cell types are especially valuable for patient classification tasks and genetic association studies. Genome-wide association study (GWAS) have pinpointed risk genes and genetic variants for complex diseases. Variants that result in a shift in gene expression (*expression quantitative trait loci*, eQTLs) offer a handle for the interpretation of disease and tissue-dependent mechanisms of gene regulation [179]. Combining GWAS insights with single-cell readouts allows to link genetic and expression variations in individual cell types, uncovering cellular regulatory circuits at unprecedented resolution. Provided sufficient cell numbers per patient within individual cell types and appropriate sequencing coverage [180], strategies are emerging to discover eQTL from single cells [181], or more often, resorting to pseudobulking cell type expression profiles. Most notably, the large single-cell datasets generated in this context have prioritized the analysis of PBMCs to identify links between risk loci of autoimmune disease and cell-type specific gene expression [182, 183]. Other studies proposed integrating genetics and single-cell readouts by combining GWAS summary statistics with individual cell types’ gene expression [184–186] or with gene programs discovered in single cells. Those approaches led to the identification of immune cell regulation pathways in a host of immune-related diseases, including COVID-19, ulcerative colitis and asthma [25, 187], but also re-confirming immune components of neurological autoimmune diseases like AD and MS [187, 188]. In the future, as large datasets of organs become available [25], it will become easier to distil the tissue-specific genetic variation that is also associated with morbidities.

## Integrative machine learning: challenges and opportunities

Multimodal integration of multiscale data collections promises a holistic approach to understanding complex disease mechanisms [174, 189–191]. Several challenges remain:

### Selection of modalities, feature extraction and interpretation

Not all modalities contribute the same quantity and quality of information to the final biological question. Without prioritization of assays and analyses, researchers may produce costly experiments to only get stuck interpreting individual omics. Furthermore, as the data increases in size and complexity, the “curse of dimensionality” becomes a serious threat to modelling and thus impedes efficiently leveraging multimodal datasets [192, 193]. A hypothesis-driven approach would help scientists formulate and prioritize central questions, enabling them to define the relevance of each modality for the focal point of research [194]. Therefore, integration methods that allow quantifying and controlling for the contribution of individual modalities to the shared latent embedding may provide an intuitive framework for prioritizing assays. Feature extraction from individual omics is a way of selecting informative features, such that redundant features’ information and noise can be minimized. Statistical tests to rank the importance of the features should take into account the dependencies between assays or are adjusted for multiple testing across the different modalities [195, 196]. Approaches like WNN [70] and TotalVI [96] although developed for paired integration include ways of quantifying the contribution of each modality to the final cell type prediction, either by associating weights to individual cells in each modality or directly quantifying how much variation is retained in joint latent representation.

Combining traditional factor models with DL methods allows to dissect the impact of covariates of interest on the individual modalities [197]. Methods that adopt mechanisms such as attention [198] or Shapley Values [199] to define features, modalities and cell types contributing to biologically relevant pathways will effectively provide a more interpretable framework to enable biomarker discovery.

#### Generating a common reference

The exponential growth of published references resulted in a babel of annotations and nomenclatures. The lack of shared annotation systems still represents a major drawback for immunologists and is effectively hindering the full exploitation of these complex datasets to identify actionable targets for study and therapy.

Building multimodal references may finally provide the framework to advance computational immunology, speeding up the process of cell annotation [22, 23, 200, 201]. Datasets that include flow or mass cytometry assays should be included in such multimodal references, to generate a resource that experienced immunologists will trust and computational immunologists can build upon. Similarly, deposited references generated by integrating single-cell proteomics and transcriptomics assays can be queried with any new unimodal dataset sharing at least a subset of the features [77, 78], without the need for time-consuming independent analysis of the new data.

#### Data infrastructure

Multimodal datasets are heterogeneous, including sparse or dense matrices, images and genomic regions, and after processing, alternative views of the data like dimensionality reduction and relational data such as graphs and ligand-receptor connections can be generated. These modalities require collecting all information in one container which allows fast access across the different layers and links the coupled modalities by their respective handle (a cell, or a patient sampled across modalities) (Fig. 2B). Data management infrastructures are emerging that respond to this need [202–206], and we anticipate they will define the foundational core for ML-integrative approaches moving forward. Finally, pipelines leveraging multimodal data containers [207, 208] offer a systematic approach for both customization and reproducibility and will speed up data processing while ensuring a stable foundation for new scientific discoveries.

#### Multimodal data, multidisciplinary teams

Generation, integration and interpretation of multimodal data calls for a diversity of expertise ranging from sample collection, data processing, storage and finally data analysis [209]. Multidisciplinary teams can tackle these challenges in a multitude of ways because they can count on the rich background of unique team members. Beyond the obvious advantages, building a multidisciplinary team takes time and careful consideration, dedicated support and infrastructure [210, 211] and requires creating a culture where individual experts can feel comfortable in sharing their expertise with colleagues of different disciplines [212].

Integrating multimodal, multiscale data collections has emerged as an effective approach to address complex disease mechanisms, inform the prioritization of assays and speed up the process of biomarker discovery and cell annotation, ultimately facilitating the identification of actionable targets for study and therapy.

## Conclusions and future directions

Using multimodal technologies, immunologists have gathered a wealth of multiscale measurements. ML approaches to integrate and interpret these data will be instrumental to understanding the mechanisms sustaining the fine regulation of the immune system in health and disease. To ensure that a vast audience of computational immunologists can benefit from these methods, it will be essential for developers to ensure the interpretability of the methods, availability of benchmarks across a wealth of conditions and well-documented use cases. Linear models will often be the preferred choice for their intuitive interpretation, especially in low-sample regimens. When data is not a limiting factor, DL and, more recently, generative AI methods, a new class of DL models with unprecedented abilities to generate new data that mimics real-world distributions, have started to show promise in numerous fields beyond their initial applications in image and text generation. These models have already proven powerful with traditionally complex tasks in immunological research, including the unbiased classification of the adaptive immune cells [89, 110–112] using positional sequence modelling [213, 214], protein structure prediction [215, 216], gene expression prediction from DNA sequences [217] and forecasting viral escape for pandemic preparedness [218]. In the future, these methods will allow to generate synthetic models of immune system behaviour under various conditions, offering insights for potential therapeutic interventions without the need for extensive laboratory experiments. Integrative methods will inform the design of new multiscale datasets, including environmental and lifestyle factors measurements, to study the role of the immune system in complex multifactorial diseases. In this outlook, computational immunologists will be pivotal in advancing scientific progress by leveraging integrative approaches to develop personalized immunological interventions. This includes the design of vaccines, immunotherapies and treatment plans that are finely tuned to individual immune system profiles, thereby enhancing the precision and effectiveness of medical treatments. With this review, we have offered a short overview of the emerging challenges and opportunities of multimodal integration applied to multiscale datasets. Recent scientific successes have started to reward those who invested in generating multimodal datasets, engineering software and fostering collaboration in multidisciplinary teams. We expect that the growing body of research on this topic will empower researchers and encourage many others to embrace the multimodal revolution.

### Supplementary Information

Additional file 1: Table S1. Integration methods cited in the review.

## Data Availability

Not applicable.
